# On the Origin of Uterine Flexions

**Published:** 1860-06

**Authors:** 


2. *On the Origin of Uterine Flexions.* By PROF. VIRCHOW. This writer sums up thus: At the seat of infraction there is found no primary alteration of tissue. Simple relations of pressure, distinguished from actual tumors, cause no anteflexions, but mostly retroflexions. Filling of the bladder and rectum cause distinct changes of position of the uterus. The changes are no longer possible when the body of the uterus is fixed at a certain height. In original shortening of a lateral ligament, there are found in children only lateral dislocation and inflexion; in persons beyond puberty, anteflexions. Anteflexions are more frequent in normal, retroflexions in pathological condition of the uterine walls.

Hence, he draws the following therapeutical deductions: In the history of flexions there is a period of simple predisposition, one of simple flexion, and one of flexion complicated with various inflammatory processes; the predisposition is frequently given by partial forms of peritonitis, which appear with colicky attacks, and are apparently very difficult to mitigate; long retention of urine and fæces favor the formation of flexions, especially at the menstrual period, that of child-bearing, etc. enlargements of the uterus, especially when united with relaxation, quickly cause flexions, and the removal of these may materially alleviate them; hence, the antiphlogistic treatment of uterine catarrh, and most careful watching of the menstrual and puerperal processes are necessary; a complete removal of anteflexion seems in the highest degree doubtful, while in retroflection it may be expected. When flexion is connected with consecutive processes, as endo- and peri-metritis, a careful local treatment is necessary.

Virchow remarks upon the views of Rokitansky, as given in the preceding article, and does not regard atrophy at the seat of infraction as primary or essential, for in anteflexions of infantile and maiden subjects there is not the slightest alteration of the uterine wall to be found. He does not consider the anatomical description of Rokitansky as in all points correct; thus, the mucous membrane of the cervical canal cannot be called callous, for it is here relatively thin; it more resembles granulation tissue. The fibro-muscular tissue of the uterus is found as well in the body as in the neck; it contains more muscular fibres and vessels in the body, and more fibrous connective tissue in the neck. Toward the mucous membrane the muscular fibres in both places cease, and there is found a distinct, apparently thick, but in the normal state by no means thick, submucous layer.—*Nashville Jour. of Med. and Surg.*, Feb. 1860.

